# Efficacy of an implantable cardioverter-defibrillator in patients with diabetes and heart failure and reduced ejection fraction

**DOI:** 10.1007/s00392-019-01415-z

**Published:** 2019-01-28

**Authors:** Rasmus Rørth, Pooja Dewan, Søren Lund Kristensen, Pardeep S. Jhund, Mark C. Petrie, Lars Køber, John J. V. McMurray

**Affiliations:** 1 British Heart Foundation Cardiovascular Research Centre, University of Glasgow, 126 University Place, Glasgow, G12 8TA UK; 2 Rigshospitalet Copenhagen University Hospital, Copenhagen, Denmark

**Keywords:** Implantable cardioverter-defibrillator, Heart failure with reduced ejection fraction, Diabetes, Sudden cardiac death

## Abstract

**Background:**

The effect of implantable cardioverter-defibrillator (ICD) therapy in patients with heart failure with reduced ejection fraction (HFrEF) and diabetes is not fully elucidated.

**Methods:**

We examined the effect of ICD therapy on sudden cardiac death, cardiovascular death and all-cause mortality, according to diabetes status at baseline in the Sudden Cardiac Death in Heart Failure Trial (SCD-HeFT). The outcomes were analyzed by use of cumulative incidence curves and Cox regressions models.

**Results:**

Of the 1676 patients randomized to an ICD or placebo, 540 (32%) had diabetes at baseline. Patients with diabetes were slightly older (61 vs 58 years) and were more often in NYHA class III (37% vs 28%). ICD therapy did not reduce the risk of sudden cardiac death in HFrEF patients with diabetes (HR = 0.85; 95% CI 0.52–1.40); even though these patients had a higher risk of sudden cardiac death compared to patients without diabetes (HR = 1.73 95% CI 1.22–2.47). By contrast, ICD therapy did reduce sudden cardiac death in HFrEF patients without diabetes (HR = 0.26; 95% CI 0.15–0.46); *P*_interaction_=0.002. The findings for cardiovascular and all-cause death were similar.

**Conclusion:**

ICD therapy did not reduce the risk of sudden cardiac death (or, as a consequence, all-cause death) in HFrEF patients with diabetes. Conversely, an ICD reduced the risk of sudden death in patients without diabetes, irrespective of etiology.

**Electronic supplementary material:**

The online version of this article (10.1007/s00392-019-01415-z) contains supplementary material, which is available to authorized users.

## Introduction

Patients with heart failure, reduced ejection fraction (HFrEF) and diabetes have a higher risk of adverse cardiovascular outcomes compared to HFrEF patients without diabetes [1–3]. This elevated risk includes a higher rate of sudden, presumed arrhythmic, cardiac death [4, 5]. Use of an implantable cardioverter-defibrillator (ICD) has been shown to reduce the risk of sudden cardiac death, and overall mortality, in patients with HFrEF. However, there is less certainty about the benefit of an ICD in patients with a non-ischemic etiology compared to those with an ischemic etiology [6, 7]. Patients with an ischemic etiology have a higher risk of sudden death than those with a non-ischemic etiology [6, 7].The effect of ICD therapy in HFrEF patients with diabetes, with and without underlying ischemic heart disease, has not been described, although this is clearly an important question given the frequent overlap between HFrEF and diabetes, the potentially different response to therapy in HFrEF patients with diabetes and the especially high risk of sudden death faced by individuals with both diabetes and an ischemic etiology. We examined these questions in the Sudden Cardiac Death in Heart Failure Trial (SCD-HeFT), the single large trial which demonstrated the benefit of this therapy in patients with HFrEF due to both ischemic and non-ischemic causes.

## Methods

### Study design and population

The design and primary results of SCD-HeFT are published [6]. In brief, in SCD-HeFT, 2521 patients in New York Heart Association (NYHA) functional class II or III, with a left ventricular ejection fraction (LVEF) ≤ 35%, were randomly assigned to placebo, amiodarone or ICD therapy in a 1:1:1 ratio. ICD therapy, but not amiodarone, reduced the primary endpoint of all-cause death. We used the public-use copy of the SCD-HeFT database obtained from the National Heart, Lung, and Blood Institute which sponsored the trial.

### Definition of diabetes and etiology

For this analysis, patients were categorized according to whether they had a history of diabetes/were treated with glucose-lowering drugs at baseline or not. Ischemic or non-ischemic etiology was determined by the investigators.

### Outcomes

The primary endpoint of this study was that used in SCD-HeFT, i.e., all-cause mortality. Cardiovascular death, sudden cardiac death, and death due to heart failure were evaluated as secondary outcomes. Outcomes were evaluated according to HF etiology, NYHA class, and estimated glomerular filtration rate (eGFR)

### Statistical analysis

Baseline characteristics according to diabetes status were described using proportions for categorical variables and mean values with standard deviations or medians with quartiles for continuous variables. Differences in baseline characteristics according to diabetes status were tested by use of χ^2^-test for categorical variables and ANOVA or Kruskal–Wallis’s test for continuous variables. Changes in LVEF were summarized by use of medians with quartiles and differences evaluated by use of quantile regression adjusted for baseline values. Kaplan–Meier curves for all-cause mortality and cumulative incidence curves for cardiovascular death and sudden cardiac death were estimated and differences between groups were compared by log rank and Gray’s test, respectively. Cox proportional hazard models were used to compare the risk according to diabetes status for all death-related outcomes. The adjusted Cox regression models included information on age, gender, randomization to ICD implantation, NYHA class, LVEF, duration of heart failure, heart failure etiology, systolic blood pressure, creatinine, history of atrial fibrillation, stroke and pulmonary disease. Log [-log(survival)] curves were used to evaluate the proportional hazard assumption. Diabetes status, heart failure etiology and NYHA class were tested for interactions with randomization to ICD implantation in relation to all outcomes. The interaction of eGFR and treatment effect according to diabetes status on all-cause mortality was analyzed by multivariable fractional polynomials with extension for interactions.

*P* values < 0.05 were considered significant. Analyses were performed by Stata version 15 and R version 3.3.2.

## Results

### Baseline characteristics

Of the 1676 patients randomized to an ICD or placebo, 540 (32%) had diabetes at baseline. Patients with diabetes were slightly older (61 vs 58 years), were more often in NYHA class III (37% vs 28%), had worse kidney function (median eGFR: 68 vs 71 mL/min per 1.73 m^2^), and higher weight (89 vs 84 kg), compared to those without diabetes (Table 1). History of hypertension (68% vs 50%), myocardial infarction (76% vs 71%), stroke (8% vs 6%) and treatment with diuretics (89% vs 81%) was more common among patients with diabetes. These findings remained consistent when patients in the amiodarone arm of the trial were added (Supplementary table 1).

**Table 1:** Baseline characteristics of patients in the placebo and ICD group

	No diabetes	Diabetes	*P* values
Patients, *n* (%)	1136 (68)	540 (32)	
Age, median [Q1–Q3]	58 [50 68]	61 [53, 68]	0.0095
Men, *n* (%)	883 (78)	411 (76)	0.4606
White, *n* (%)	890 (78)	393 (73)	0.0218
ICD implantation, *n* (%)	570 (50)	259 (48)	0.3971
NYHA class III, *n* (%)	314 (28)	202 (37)	0.0001
Ischemic heart failure etiology, *n* (%)	551 (49)	333 (62)	0.0001
Left ventricular ejection fraction, median [Q1–Q3]	23.5 [19, 30]	25 [20, 30]	0.0074
Systolic blood pressure, mmHg, median [Q1–Q3]	117 [104, 130]	120.0 [108.0, 135.0]	0.0011
Diastolic blood pressure, mmHg, median [Q1–Q3]	70 [62, 80]	70 [60, 80]	0.3732
Weight, Kg, median [Q1–Q3]	84 [73, 98]	89 [77, 104]	0.0001
Heart rate, beats/min, median [Q1–Q3]	72 [64, 83]	77 [68, 86]	0.0001
eGFR, mL/min/1.73 m^2^, median [Q1–Q3]	71 [57, 85]	68 [52, 82]	0.0007
Medical history, *n* (%)			
Atrial fibrillation/flutter	170 (15)	88 (16)	0.4803
Hypertension	564 (50)	367 (68)	0.0001
Myocardial infarction	478 (71)	282 (76)	0.0402
Stroke	64 (6)	45 (8)	0.0362
Pulmonary disease	213 (19)	120 (22)	0.0959
Medication^a^, *n* (%)			
ACE inhibitor or ARB	1093 (96)	517 (96)	0.641
Β-Blocker	788 (69)	369 (68)	0.6691
Mineralocorticoid-receptor antagonist	210 (19)	107 (20)	0.5162
Diuretic	923 (81)	480 (89)	0.0001
Digoxin	763 (67)	378 (70)	0.2447
Insulin	0 (0)	198 (37)	–
Oral hypoglycemic agents	0 (0)	325 (60)	–

Q-quartile, *ICD* implantable cardioverter defibrillator, *ACE* angiotensin-converting enzyme, *ARB* angiotensin-receptor blocker, *eGFR* estimated glomerular filtration rate

^a^At baseline

### Complications during ICD implantation

Patients with diabetes had similar risk of ICD-related infections and a lower risk of bleeding and pneumothorax than patients without diabetes. Specifically, infection complicated implantation in 20 (3.5%) patients without diabetes, compared to 9 (3.1%) patients with diabetes (*p* = 0.76); the respective numbers for bleeding were 29 (5.1%) vs. 4 (1.5%), *p* = 0.02 and for pneumothorax 9 (1.6%) vs. 0 (0%), *p* = 0.04.

### Change in left ventricular ejection fraction after randomization

Patients with diabetes had a slightly but significantly higher LVEF at baseline, compared to patients without diabetes, and also marginally but significantly greater increase in LVEF during follow-up: median change from baseline to 12 months (*n* = 1159) + 4 [IQR 0.0, 12.5] in patients without diabetes and + 5 [− 1.0, 12.5] in patients with diabetes, *p* = 0.004; the corresponding changes at 30 months (*n* = 777) were: + 5 [0.0, 15.0] vs + 6 [0.0, 16.0], respectively, *p* = 0.005. As a result, 32.5% of patients with diabetes (compared to 30.0% without diabetes) had a LVEF > 35% after approximately 12 months of follow-up and 40% (compared with 36%) at 30 months.

### Clinical outcomes according to diabetes status

During a median follow-up of 46 months (Q1-Q3:35–55), sudden cardiac death occurred in 63 (12%) patients with diabetes and 71 (6%) without. Death from cardiovascular causes occurred in 128 (24%) patients with diabetes and in 161 (14%) patients without; 187 (35%) patients with diabetes died from any cause, compared to 239 (21%) patients without diabetes. In adjusted analyses, diabetes was associated with a higher risk of sudden cardiac death (HR = 1.73 95% CI 1.22–2.47; *p* = 0.002), cardiovascular death (HR = 1.52; 95% CI 1.19–1.93; *p* = 0.001) and all-cause mortality (HR = 1.50; 95% CI 1.23–1.83; *p* < 0.0001); Table 2.

**Table 2:** Event rates and hazard ratios for all outcomes according to diabetes status of patients in the placebo and ICD group

	Events (n)	Crude rate per 100 py (95% CI)	Unadjusted HR (95% CI)	*p* values	Adjusted HR^a^ (95% CI)	*p* values
All-cause mortality						
Diabetes	187	10.8 (9.4–12.5)	1.80 (1.49–2.18)	< 0.0001	1.50 (1.23–1.83)	< 0.0001
No diabetes	239	6.1 (5.3–6.9)	1.00 (ref.)		1.00 (ref.)	
CV death						
Diabetes	128	7.4 (6.2–8.8)	1.82 (1.44–2.30)	< 0.0001	1.52(1.19–1.93)	0.001
No diabetes	161	4.1 (3.5–4.8)	1.00 (ref.)		1.00 (ref.)	
Sudden cardiac death						
Diabetes	63	3.7 (2.9–4.7)	2.02 (1.44–2.83)	< 0.0001	1.73 (1.22–2.47)	0.002
No diabetes	71	1.8 (1.4–2.3)	1.00 (ref.)		1.00 (ref.)	
Non-sudden CV death						
Diabetes	124	7.2 (6.0-8.6)	1.71 (1.36–2.16)	< 0.0001	1.42 (1.11–1.80)	0.005
No diabetes	168	4.3 (3.7–4.9)	1.00 (ref.)		1.00 (ref.)	

*HR* hazard ratio, *py* person years, *CV* cardiovascular, *ref* reference

^a^Adjusted for age, gender, randomization to ICD implantation, NYHA class, left ventricular ejection fraction, duration of HF, HF etiology, systolic blood pressure, creatinine, history of atrial fibrillation, stroke and pulmonary disease

### Effect of ICD therapy according to diabetes status

**Fig. 1:**
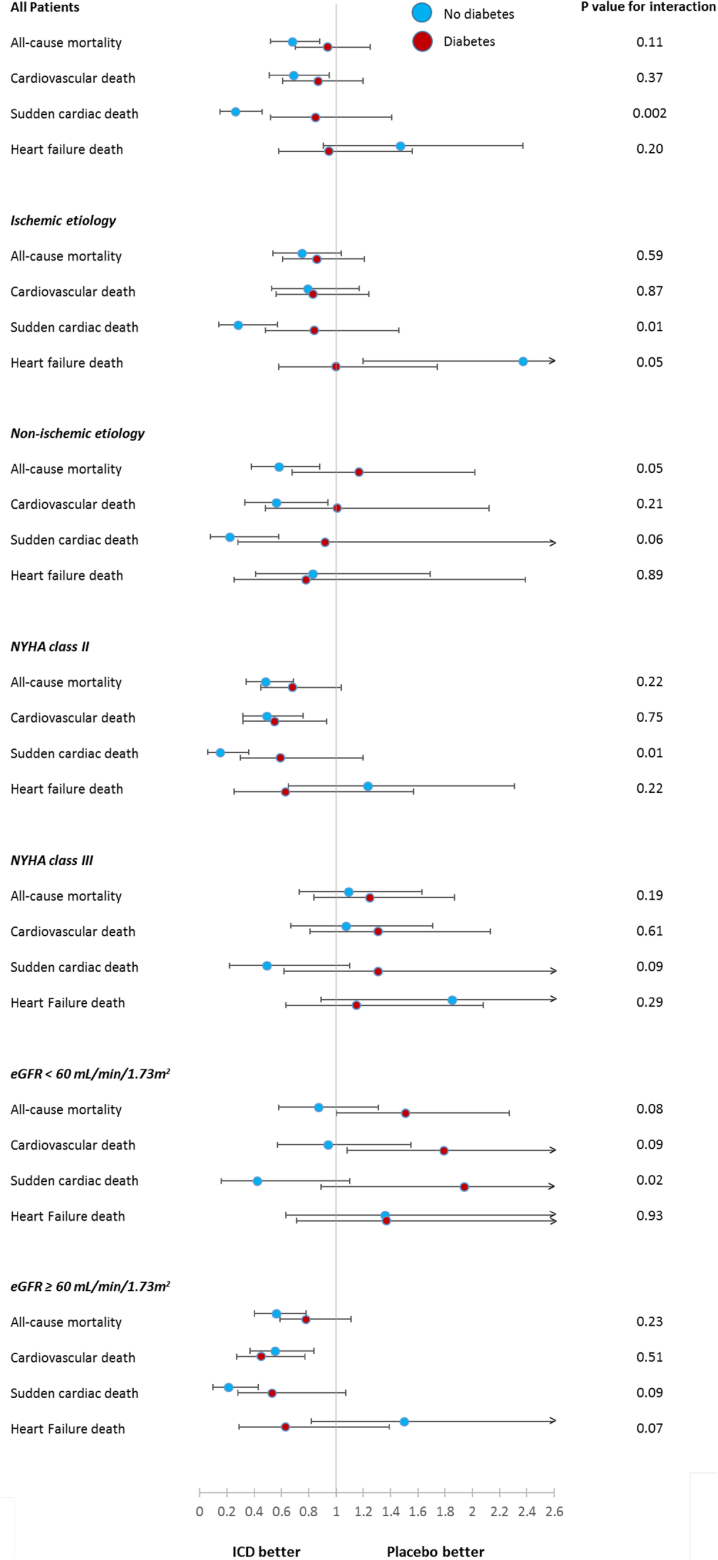
Hazard ratios for the comparison of ICD therapy vs placebo

**Fig. 2:**
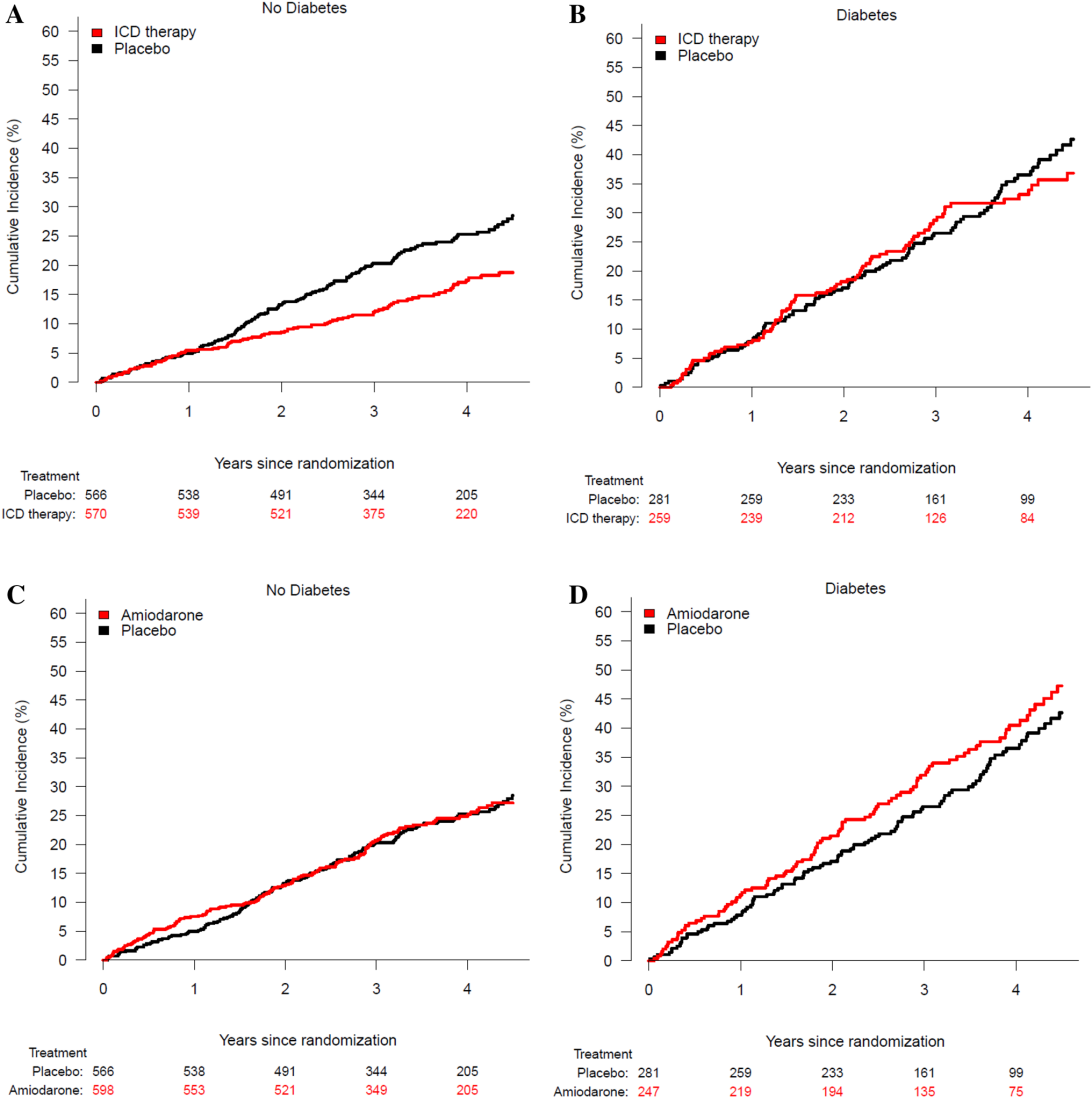
Risk of death among patients with and without diabetes

ICD therapy did not reduce the risk of sudden cardiac death in HFrEF patients with diabetes (HR = 0.85; 95% CI 0.52–1.40). By contrast, ICD therapy did reduce sudden cardiac death in HFrEF patients without diabetes (HR = 0.26; 95% CI 0.15–0.46); *P*_interaction_=0.002. The effects of ICD therapy on any cardiovascular death and death from heart failure are shown in Fig. 1. Examining all-cause mortality, 84 patients (32%) with diabetes died in the ICD group compared to 103 patients (37%) with diabetes in the placebo group; HR = 0.94; 95% CI 0.70–1.25; *p* = 0.65 (Fig. 2). Among those without diabetes, 98 patients (17%) died in the ICD group, compared to 141 patients (25%) without diabetes in the placebo group; HR = 0.68; 95% CI 0.52–0.88; *p* = 0.003 (Fig. 2), p for interaction = 0.11 (Fig. 1).

Patient level data on appropriate and inappropriate ICD discharges were not available.

### Effect of ICD implantation according to diabetes status and kidney function

**Fig. 3:**
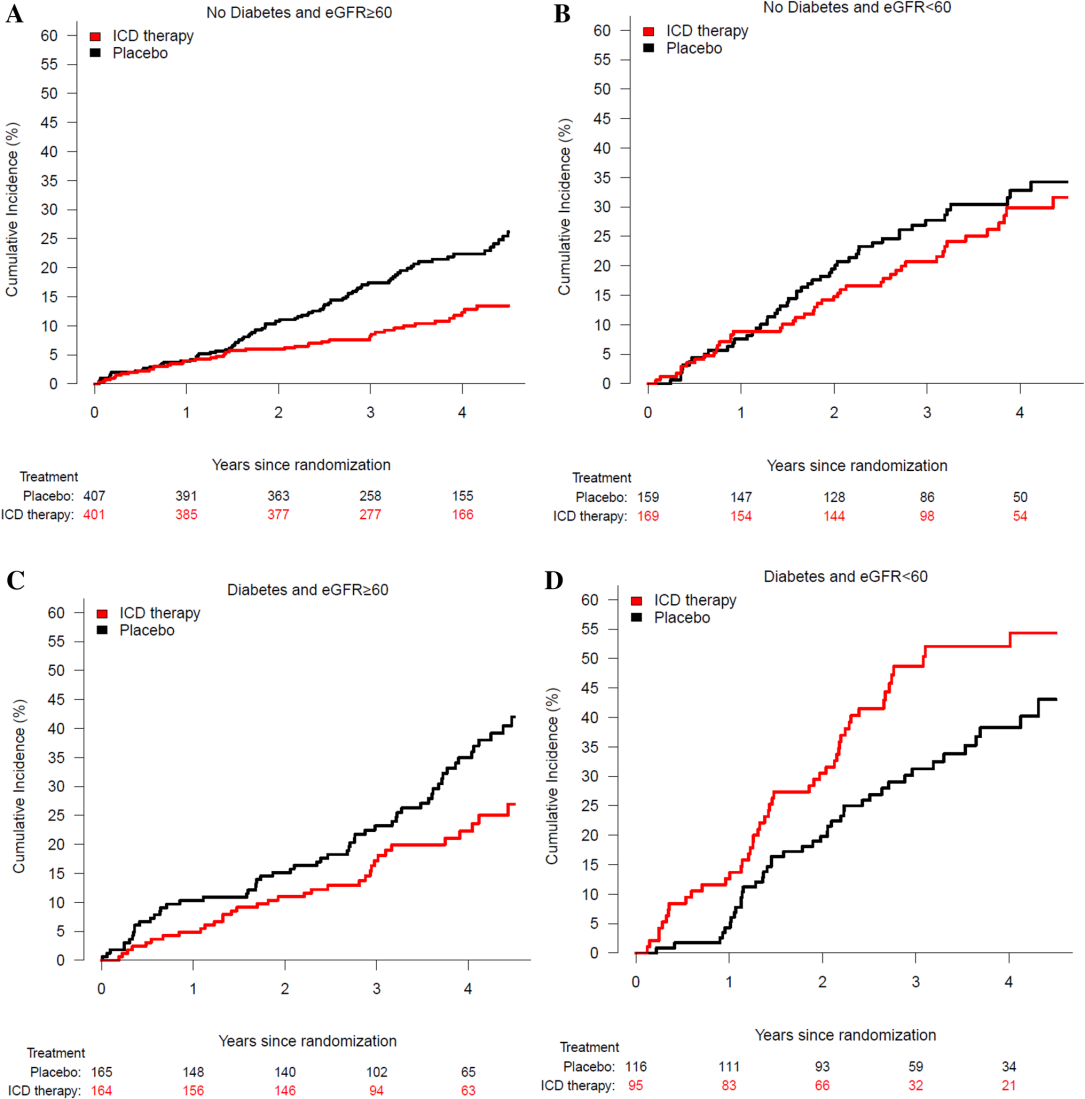
Risk of death according to diabetes status and eGFR ≥ 60 mL/min/1.73 m^2^ (A + C) or eGFR < 60 mL/min/1.73 m^2^ (B + D)

The reduction in sudden cardiac death with an ICD was apparent in patients without diabetes, irrespective of kidney function, although attenuated in no-diabetes patients with chronic kidney disease (CKD) (Fig. 3 and Supplementary Fig. 1). By contrast, the effect of ICD therapy on sudden cardiac death in patients with diabetes was absent in patients with concomitant CKD and attenuated in those with preserved renal function, compared to no diabetes patients with an eGFR > 60 mL/min/1.73 m^2^ (Fig. 3). Similar findings were observed when eGFR was examined as a continuous variable (Supplementary Fig. 1).

All-cause mortality was lower in the ICD group, compared with the placebo group, in patients without diabetes, irrespective of renal function category. By contrast, ICD treatment did not clearly reduce all-cause mortality in patients with diabetes but without CKD. In patients with both diabetes and CKD, mortality was higher in the ICD group than in the placebo group (Fig. 3).

### Effect of ICD implantation according to diabetes status and ischemic vs. non-ischemic etiology

**Fig. 4:**
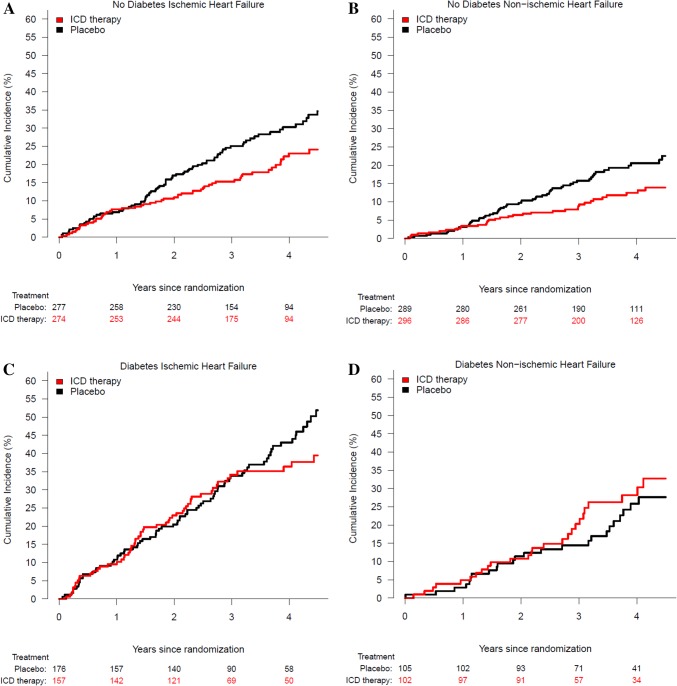
Risk of death according to ischemic (A + C) and non-ischemic (B + D) HF etiology and diabetes

The reduction in sudden cardiac death with an ICD was apparent in patients without diabetes, irrespective of etiology (Fig. 4). By contrast, there was no clear effect of ICD therapy on sudden cardiac death in patients with diabetes, whether the etiology of HFrEF was ischemic or non-ischemic (Fig. 4). The effect of ICD therapy on sudden cardiac death was significantly modified by diabetes status overall, according to etiology and CKD status and in relation to NYHA class (Fig. 1).

All-cause mortality was lower in the ICD group, compared with the placebo group, in patients without diabetes, irrespective of etiology (ischemic vs. non-ischemic). By contrast, ICD treatment did not reduce all-cause mortality in patients with diabetes, irrespective of etiology.

### Effect of amiodarone according to diabetes status

In the amiodarone group, 97 (39%) with diabetes died compared to 103 (37%) with diabetes in the placebo group; HR = 1.19; 95% 0.90–1.57; *p* = 0.22; Fig. 1. In patients without diabetes, 143 patients (24%) died in the amiodarone group compared to 141 patients (25%) without diabetes in the placebo group; HR = 1.01; 95% CI 0.80–1.27; *p* = 0.96 (Fig. 1).

### Effect of amiodarone according to diabetes status and ischemic vs. non-ischemic etiology

Overall, the effect of amiodarone, compared with placebo, did not differ according to diabetes status, etiology, NYHA class or CKD status (Supplementary Fig. 2).

## Discussion

In this retrospective subgroup analysis of SCD-HeFT, ICD therapy did not reduce the risk of sudden cardiac death, and therefore the risk of death from any cause, in HFrEF patients with diabetes although these patients were at high risk of sudden, presumed arrhythmic, death. By contrast, ICD therapy did reduce the risk of sudden cardiac death, and all-cause mortality, in HFrEF patients without diabetes. This difference in outcomes with ICD therapy was not explained by etiology (ischemic versus non-ischemic) of HFrEF.

The explanation for the lack of mortality benefit in patients with diabetes is uncertain although the issue of competing risks needs to be considered as it is more relevant with an ICD than other therapies for heart failure and more relevant in patients with diabetes than in those without. Specifically, ICD therapy only reduces one mode of cardiovascular death, leaving the competing risk of other cardiovascular, as well as non-cardiovascular causes of death. Patients with diabetes are at greater risk of non-cardiovascular death than patients without diabetes, limiting the impact of any cardiovascular treatment on overall mortality. While the latter was true even in the relatively young patients enrolled in SCD-HeFT, there was only a small excess of non-cardiovascular death among individuals with diabetes. However, and more importantly, there was no evidence of a bidirectional effect of ICD therapy on different modes of cardiovascular death in patients with diabetes. Quite simply, ICD treatment was of no benefit in patients with diabetes because this therapy had no effect on sudden cardiac death (and the benefit of device therapy in patients without diabetes was attributable to the reduction in sudden cardiac death with an ICD). There was no suggestion that ICD therapy increased non-sudden cardiovascular death, particularly death from heart failure, in patients with diabetes (whereas there was some indication of this in patients without diabetes).

Because of recent debate about the value (or lack of value) of ICDs in patients with non-ischemic etiology, we also examined the interaction between diabetes status and etiology (ischemic versus non-ischemic) and effect of ICD therapy on outcomes. We found that an ICD reduced the risk of sudden death in patients without diabetes, irrespective of etiology. In patients with diabetes, ICD therapy had no effect on sudden death, and this lack of benefit was true for both ischemic and non-ischemic etiologies. This finding is a particularly concerning for HFrEF patients with the relatively common combination of diabetes and an ischemic etiology, as they are at especially high risk of sudden cardiac death.

Assuming our findings are correct, they raise the possibility that ICDs are less effective at aborting ventricular arrhythmias in HFrEF patients with diabetes or, secondly, that a substantial proportion of adjudicated sudden cardiac deaths in HFrEF patients with diabetes are caused by mechanisms other than ventricular arrhythmias. Regarding the first possibility, comparison with the Multicenter Automatic Defibrillator Implantation Trial II (MADIT II), the pivotal ICD trial in patients after myocardial infarction is instructive [8]. In MADIT II, ICD treatment appeared to reduce all-cause mortality by a similar amount in patients with (*n* = 433) and without diabetes (*n* = 798) [9]. In other words, it was possible to show that ICDs are of benefit in patients with diabetes in a related, although not identical, population. If we assume most sudden deaths in chronic heart failure are caused by ventricular arrhythmias, the lack of benefit of an ICD in HFrEF patients (compared to those with myocardial infarction) suggests that development of the syndrome of symptomatic heart failure, weeks, months or years after infarction, is associated with changes in the diabetic heart, such as deposition of glycoproteins, extracellular matrix accumulation, interstitial edema, myocyte hypertrophy and myocyte loss, that attenuate the effect of electrical therapy [10, 11]. Alternatively, the proportion of ventricular arrhythmias arising because of acute ischemia is likely to be less in chronic HFrEF than after myocardial infarction, given the much higher rate of occurrence of acute coronary syndromes in high-risk infarct survivors, compared with chronic HFrEF patients [12–17]. A further potential effect-modifier is renal function. The benefit of an ICD is known to be reduced in patients with renal impairment and HFrEF patients with diabetes have worse renal function than HFrEF patients without diabetes [18]. We observed that SCD-HeFT patients with both HFrEF and diabetes were at especially high risk and appeared to get no benefit at all from an ICD (indeed, among these patients, those with a device did worse than those without a device). However, even in patients with preserved kidney function, the benefit of an ICD was less in patients with diabetes than in those without.

Another relevant factor may be the considerable increase in LVEF over time in patients in SCD-HeFT, an increase which was slightly but significantly greater in patients with diabetes (+ 5% at 1 year), compared to those without (+ 4%). By a year after randomization, 31% patients with diabetes had increased their LVEF to > 35% compared to 27% of those without diabetes. Although these differences were relatively small (and confounder by survivor bias), they may also have contributed to a lower risk of arrhythmias and less potential for benefit from an ICD in patients with diabetes.

Alternatively, a significant proportion of sudden cardiac deaths in HFrEF patients with diabetes, may not be caused by ventricular arrhythmias. In SCD-HeFT, the ICDs used were single chamber systems programmed only to shock and not to pace. Consequently, these devices did not protect against bradycardia-related events.

Another potential contributor to lack of overall benefit of ICD therapy in patients with diabetes, compared to those without, might be a greater complication rate during device implantation in individuals with diabetes, resulting in less net benefit. However, we found that the complication rate was somewhat less in patients with diabetes, compared to those without, at least in the short-term (and the overall number of complications was small). A recent pooled analysis of 4 primary prevention ICD trials, including SCD-HeFT, also reported this trend [19].

Lastly, there is the possibility that some sudden deaths in HFrEF patients with diabetes are not related to a primary cardiac arrhythmia. These may be cardiovascular in nature (such as stroke) or due to other causes such as hypoglycemia; here it is notable that 37% of patients with diabetes in SCD-HeFT were treated with insulin at baseline [20, 21].

One other finding in this study is worthy of comment. Amiodarone therapy, which was ineffective overall, led to a numerically higher death rate in patients with diabetes, when compared to placebo. Amiodarone did not increase mortality, compared with placebo, in patients without diabetes.

### Limitations

This is a post hoc analysis and, as such, warrants caution in the interpretation of the results. One concern is the relatively small number of patients with diabetes (*n* = 540). However, the number of patients with diabetes in MADIT II was smaller and yet that trial showed a benefit of ICD therapy, suggesting that our finding of no effect was not due to lack of power [9]. A recent pooled analysis of four primary prevention trials also found no overall benefit of ICD therapy in patients with diabetes but that analysis mixed patients with HFrEF and myocardial infarction, populations with different risks of sudden and non-sudden cardiovascular death and potentially different mechanisms underlying sudden cardiac death [19]. That report did not describe the effect of ICD therapy on sudden cardiac death versus non-sudden cardiovascular death, which is crucial in understanding potentially differential effects of this treatment, i.e., an ICD can only reduce all-cause mortality by reducing sudden cardiac death (substantially) without simultaneously increasing the risk of non-sudden death.

We also did not have data on rates of device therapy and whether this was appropriate or inappropriate. However, another trial, the Multicenter Automatic Defibrillator Implantation Trial–Reduce Inappropriate Therapy trial (MADIT-RIT), demonstrated less inappropriate therapy in patients with diabetes compared to those without [22].

## Conclusion

ICD therapy did not reduce the risk of sudden cardiac death (or, as a consequence, all-cause death) in HFrEF patients with diabetes. Conversely, an ICD reduced the risk of sudden death in patients without diabetes, irrespective of etiology. This may be because ICDs are less effective at aborting ventricular arrhythmias in HFrEF patients with diabetes or that some episodes of sudden death in these patients may be caused by other cardiac mechanisms (e.g., bradycardia) or non-cardiac events (e.g., hypoglycaemia).

## Electronic supplementary material

Below is the link to the electronic supplementary material.

Supplementary material 1 (DOCX 2328 KB)
