# Publisher Correction to: Efficacy of an implantable cardioverter-defibrillator in patients with diabetes and heart failure and reduced ejection fraction

**DOI:** 10.1007/s00392-019-01582-z

**Published:** 2020-01-16

**Authors:** Springer Berlin Heidelberg

**Affiliations:** http://www.springer.com/392

## Publisher Correction to: Clinical Research in Cardiology (2019) 108:868–877 10.1007/s00392-019-01415-z

The article was initially published with incorrect copyright information or changed later to incorrect copyright information.

Upon publication of this Correction, the copyright of the article changed to “The Author(s)”.

The original article has been corrected.

