# Supplementary material for: Efficacy of an implantable cardioverter-defibrillator in patients with diabetes and heart failure and reduced ejection fraction
Source: Clin Res Cardiol. 2019 Jan 28;108(8):868–77. doi: 10.1007/s00392-019-01415-z (PMC6652172; doi:10.1007/s00392-019-01415-z)

**Supplementary table 1:** Baseline characteristics of all patients (control, amiodarone and ICD groups combined)

|  | **No diabetes** | **Diabetes** | **P-values** |
| --- | --- | --- | --- |
| Patients, n (%) | 1734 (69) | 787 (31) |  |
| Age, median [Q1-Q3] | 59.0 [50.0, 68.0] | 61.0 [53.0, 68.0] | 0.0037 |
| Men, n (%) | 1333 (77) | 600 (76) | 0.7267 |
| White, n (%) | 1365 (79) | 567 (72) | 0.0002 |
| **Treatment groups, n (%)** |  |  | 0.2158 |
| Placebo | 566 (33) | 281 (36) |  |
| ICD implantation | 570 (33) | 259 (33) | 0.3971 |
| Amiodarone | 598 (34) | 247 (31) |  |
| NYHA class III, n (%) | 466 (27) | 294 (37) | 0.0001 |
| Ischemic heart failure etiology, n (%) | 835 (48) | 475 (60) | 0.0001 |
| Left ventricular ejection fraction, median [Q1-Q3] | 23.5 [19, 30] | 25 [20, 30] | 0.0001 |
| Systolic blood pressure, mmHg, median [Q1-Q3] | 118 [104, 130] | 120 [108, 136] | 0.0001 |
| Diastolic blood pressure, mmHg, median [Q1-Q3] | 70 [62, 80] | 70 [60, 80] | 0.1821 |
| Weight, Kg, median [Q1-Q3] | 84 [73, 97] | 90 [77, 104] | 0.0001 |
| Heart rate, beats/min, median [Q1-Q3] | 72 [64, 83] | 76 [66, 85] | 0.0001 |
| eGFR, mL/min/1.73 m^2^, median [Q1-Q3] | 71 [57, 85] | 67 [52, 82] | 0.0001 |
| **Medical history**, n (%) |  |  |  |
| Atrial fibrillation/flutter | 268 (15) | 122 (16) | 0.9762 |
| Hypertension | 866 (50) | 534 (68) | 0.0001 |
| Myocardial infarction | 712 (70) | 406 (76) | 0.0112 |
| Stroke | 109 (6) | 57 (7) | 0.3695 |
| Pulmonary disease | 311 (18) | 169 (21) | 0.036 |
| **Medication***, n (%) |  |  |  |
| ACE inhibitor or ARB | 1676 (97) | 756 (96) | 0.4538 |
| Β-blocker | 1208 (70) | 530 (67) | 0.2432 |
| Mineralocorticoid-receptor antagonist | 324 (19) | 160 (20) | 0.3311 |
| Diuretic | 1406 (81) | 702 (89) | 0.0001 |
| Digoxin | 1189 (69) | 566 (72) | 0.0902 |
| Insulin | 0 (0) | 293 (37) | - |
| Oral hypoglycemic agents | 0 (0) | 477 (61) | - |

Abbreviations: Q-quartile, ICD- implantable cardioverter defibrillator,

ACE - angiotensin-converting enzyme, ARB - angiotensin-receptor blocker,

eGFR - estimated glomerular filtration rate,

* at baseline

**Supplementary figure 1:** Treatment effect of ICD implantation on all-cause mortality across the spectrum of eGFR according to diabetes status

A:


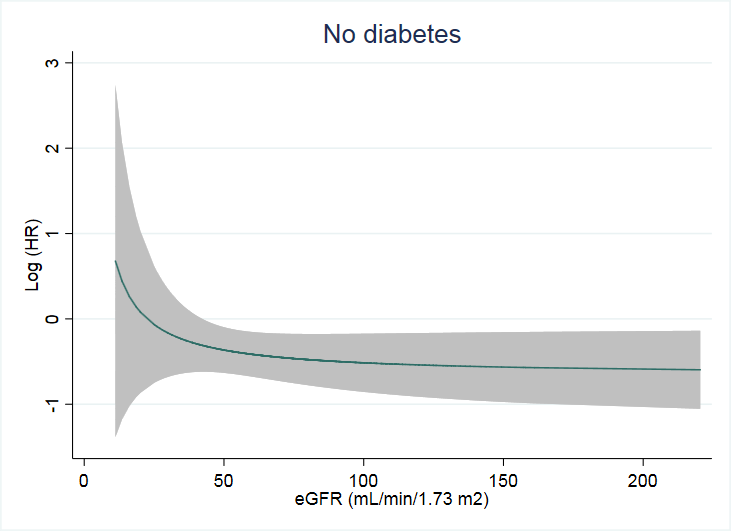


B:


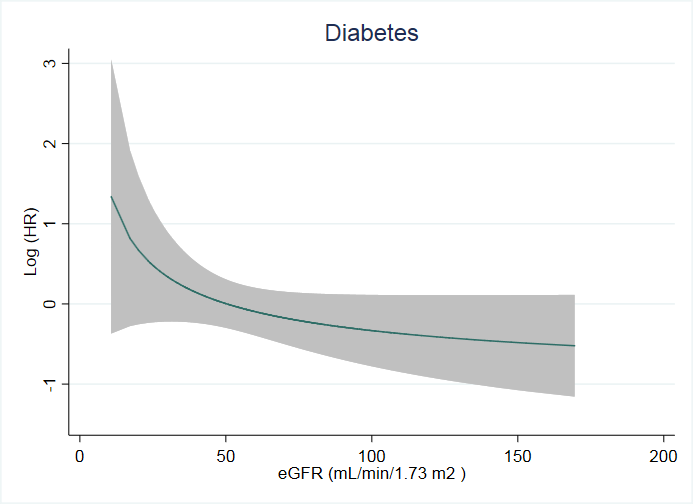


**Supplementary figure 2:** Hazard ratios for the comparison of amiodarone versus placebo
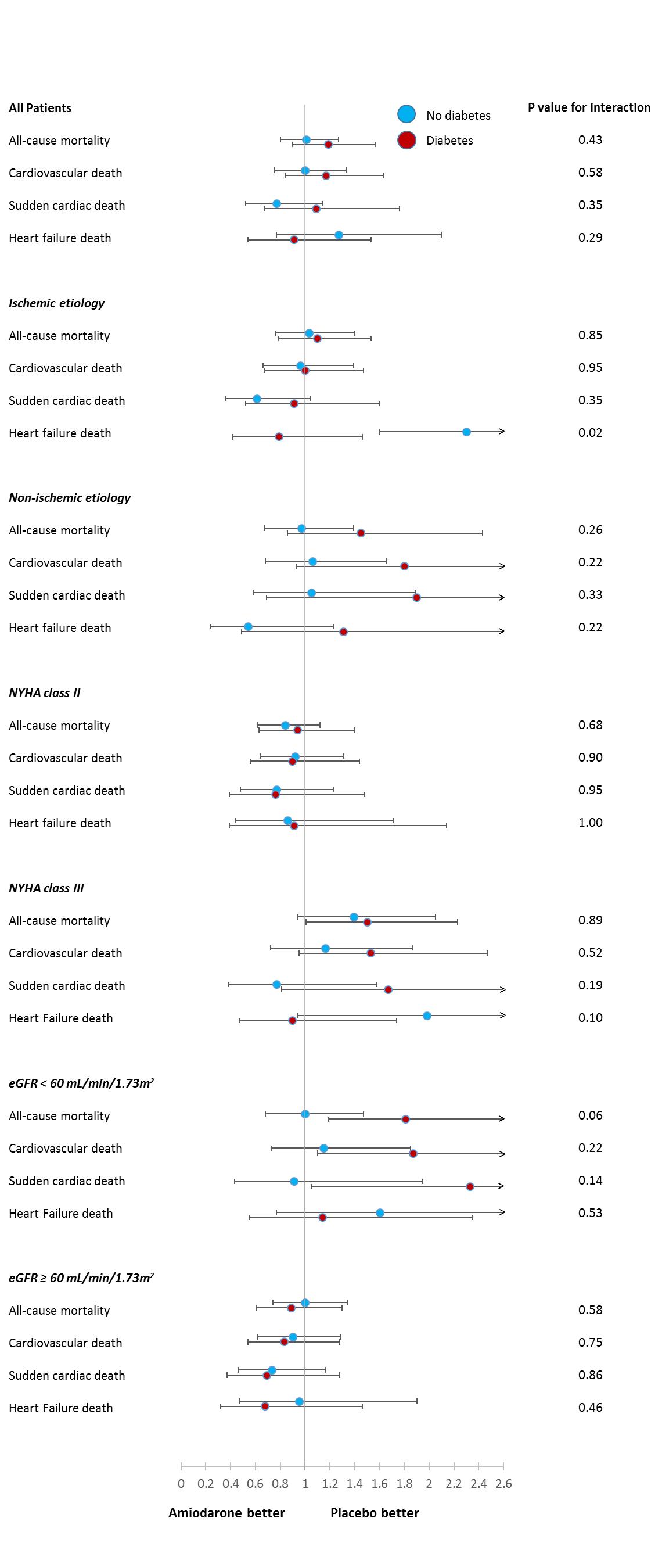

Supplement: Supplementary file 1 — Supplementary material 1 (DOCX 2328 KB) [file 392_2019_1415_MOESM1_ESM.docx]
